# Economic analysis of hemodialysis and urgent-start peritoneal dialysis therapies

**DOI:** 10.1590/2175-8239-JBN-2024-0051en

**Published:** 2025-01-10

**Authors:** Alexandre Minetto Brabo, Dayana Bitencourt Dias, Everton Nunes da Silva, Daniela Ponce

**Affiliations:** 1 Universidade Estadual Paulista, Faculdade de Medicina, Departamento de Clínica Médica, Botucatu, SP, Brazil.; 2 Universidade de Brasília, Departamento de Saúde Coletiva, Brasília, DF, Brazil.

**Keywords:** Peritoneal Dialysis, Hemodialysis, Health Economic Evaluation, Cost-Effectiveness Evaluation, Cost-Efficiency Analysis, Health Policy, Evidence-Informed Policy, Diálise Peritoneal, Hemodiálise, Avaliação Econômica em Saúde, Avaliação de Custo-Efetividade, Análise Custo-Eficiência, Política de Saúde, Política Informada por Evidências

## Abstract

**Introduction:**

Unplanned initiation of renal replacement therapy (RRT) in chronic kidney disease (CKD) patients is a common situation worldwide. In this scenario, peritoneal dialysis (PD) has emerged as a therapeutic option compared to hemodialysis (HD). In planned RRT, the costs of PD are lower than those of HD; however, the literature lacks such analyses when initiation is urgent.

**Objective:**

To clinically and economically evaluate, from the perspective of the Unified Health System (SUS, *Sistema Único de Saúde*), the strategy of initiating unplanned RRT using HD or PD in patients during their first year of therapy.

**Methodology:**

Quasi-experimental study with cost-effectiveness analysis based on primary data from incident patients on RRT, over a twelve-month follow-up period, using the intention-to-treat approach. Data collection occurred prospectively, directly from medical records, computing data on the use of dialysis therapy, high-cost medications, procedures in dialysis accesses and recorded events. Costs were estimated using the amounts reimbursed by the SUS. In the economic analysis, the application of the bootstrap method and the construction of graphical representations were proposed.

**Results:**

At the end of one year, there were no differences between costs and effectiveness when initiating unplanned RRT using either PD or HD.

**Conclusion:**

Starting RRT with PD is a similar option to starting with HD in patients requiring unplanned methods. The minimal initial investment required to establish PD slots makes it a strong option as a public health policy for expanding RRT in developing countries.

## Resumo

**Introdução:**

O início da terapia renal substitutiva (TRS) de modo não planejado em portadores de doença renal crônica (DRC) é situação frequente mundialmente. Nesse cenário, a diálise peritoneal (DP) tem se evidenciado como opção terapêutica em comparação à hemodiálise (HD). Na TRS planejada, os custos da DP são inferiores aos da HD, porém a literatura carece dessas análises quando o início é urgente.

**Objetivo:**

Avaliar clínica e economicamente, na perspectiva do Sistema Único de Saúde (SUS), a estratégia de iniciar TRS não planejada por HD e DP em pacientes durante seu primeiro ano de terapia.

**Metodologia:**

Estudo quase experimental com análise de custo-efetividade a partir de dados primários de pacientes incidentes em TRS, em horizonte temporal de doze meses, por intenção de tratamento. A coleta de dados ocorreu de maneira prospectiva, diretamente dos prontuários médicos, computando dados de utilização de terapia dialítica, medicações de alto custo, procedimentos em acessos dialíticos e eventos ocorridos, com estimativa de custos pelos valores repassados pelo SUS. Na análise econômica, foi proposta a aplicação de método de *bootstrapping* e construção de gráficos.

**Resultados:**

No fim de um ano, não houve diferenças entre custos e efetividade ao iniciar a TRS de maneira não planejada por DP e HD.

**Conclusão:**

Iniciar TRS por DP é opção semelhante a iniciar por HD em pacientes com necessidade de métodos não planejados. A mínima necessidade de investimento inicial para criação de vagas de DP é fator que a fortalece como política de saúde pública para expansão da TRS em países em desenvolvimento.

## Introduction

Chronic kidney disease (CKD) is a complex health condition associated with increased morbidity and mortality, as well as high demands on health system resources^1,2,3,4^. Given the limited effectiveness of measures to control CKD progression, some patients will require renal replacement therapy (RRT).

The optimal scenario for initiating dialysis methods is within a planned framework, with prior follow-up in consultations with a multidisciplinary team, including the nephrologist, involving guidance on dialysis, lifestyle changes, and optimization of pharmacological and non-pharmacological treatments. Additionally, at the time of starting RRT, in hemodialysis (HD), the dialysis access via arteriovenous fistula should be constructed and functioning. In peritoneal dialysis (PD), the Tenckhoff catheter needs to be healed and properly functioning, the home environment must be suitable for the therapy, and caregivers or the patient should be trained to perform it^2^.

Although desirable, this is not the reality in much of the world. In Brazil, around 60% of patients do not initiate dialysis in a planned manner^5,6^. Several factors contribute to this issue, including late diagnosis, challenges in specialist referrals, and difficulties in securing permanent access early on^7,8,9,10,11^.

Unplanned dialysis, also known as urgent-start dialysis is defined as the initiation of therapy without prior consultation with a nephrologist, or if a consultation occurred, it was within less than 90 days before starting dialysis. It could be either PD or HD, with the following particularities: in HD, RRT begins without a functioning and usable arteriovenous fistula, therefore via a central venous catheter (CVC)^12^. In PD, it would be characterized by the start of therapy within 3 days of catheter implantation, without prior family training and without home preparation^13^.

In the planned scenario, the clinical outcomes between PD and HD are quite similar, particularly in the first two years of therapy^14,15^. Growing evidence in the unplanned scenario concludes that PD is also a safe and effective option. A systematic review by Zang et al.^16^ showed that patients undergoing urgent-start PD had similar infectious complications and mortality rates compared to patients treated with urgent-start HD. More recently, Ding et al.^17^, in a systematic review published in 2022, demonstrated lower mortality and lower infectious complication rates in patients treated with urgent-start PD. In addition to the conclusions of these studies, there are advantages attributed to the preservation of the vascular vessels and residual renal function, and fewer serious complications, such as bacteremia and hospitalizations^5,18,19,20,21,22^. Its implementation serves as an important tool for expanding PD programs^19^, providing an alternative to the limited availability of HD slots in the country^23,24,25^.

Despite positive outcomes, when it comes to high-cost therapies and their budgetary impact, clinical results alone are not considered sufficient to change health policies. Therefore, conducting economic analyses becomes essential. Studies published to date indicate that PD is a more cost-effective therapy than HD in the context of planned RRT^26,27,28^. These include the study by Chang et al.^27^, which evaluated the survival rate over a 14-year follow-up period, and the costs of HD *vs* planned PD therapies. Life expectancy was similar between treatments (19.2 *vs* 19.08 years). So was quality of life. The results show that PD was dominant, with savings of 5,000 dollars per quality-adjusted life year (QALY). However, excluding incident patients^27^ overlooks an important population, as morbidity and mortality are high during the first few months of therapy^4^.

In the unplanned RRT scenario, only one American study, published in 2014^29^, analyzed the costs associated with dialysis methods over the first 90 days of treatment, concluding that urgent-start PD may have lower costs (U$ 16,398 compared to U$ 19,352 for urgent-start HD).

The shortage of HD slots in Brazil, frequently reported in the media^23,24,25^, and the high cost for its expansion make PD an interesting alternative for expanding, rather than replacing, RRT, as it does not require large structures^30^ and machinery. The study is justified by the evident need for further economic analysis in unplanned RRT. Its aim is to clinically and economically evaluate, from the perspective of the SUS, the strategy of initiating dialysis methods in an unplanned manner, with a twelve-month follow-up period from the start of outpatient therapy.

## Methods

Quasi-experimental study with economic analysis through cost-effectiveness evaluation based on primary data from incident PD and HD patients at HC-FMB. Data were prospectively collected from all stage 5 CKD patients, aged 18 and over, whose dialysis treatment necessarily started in an unplanned manner between January 1, 2016, and January 1, 2018. HC-FMB is the only tertiary care service provided by the SUS in its region, and high-complexity treatments are performed exclusively at the institution. Thus, the care data recorded in medical records are complete and reliable.

The immediate indications for dialysis were uremia and refractory cases of hypervolemia, hyperkalemia, and metabolic acidosis. Absolute contraindications to PD are: recent abdominal surgery (less than 30 days); multiple previous abdominal surgeries (more than two); presence of peritoneal fibrosis or adhesions; fungal peritonitis; acute respiratory failure (FiO^2^ > 70%); abdominal wall infections; severe hyperkalemia with characteristic electrocardiographic changes, and acute pulmonary edema^31^.

### Allocation to Groups and Dialysis Procedures

Randomizing the initiation of RRT has thus far been an unsuccessful task in the literature^14^, since there are absolute contraindications to performing PD, and patient choice should always be considered.

In the absence of the aforementioned contraindications, and with the consent of the individual and family members, the patient was allocated to the PD initiation group and underwent Tenckhoff catheter implantation. The initiation of outpatient dialysis occurs at the dialysis center through intermittent PD (IPD), performed in at least three weekly daytime sessions of automated peritoneal dialysis. Adjustments were made according to clinical and laboratory assessments by the medical team, until the patient is able to maintain home dialysis therapy or another outcome is reached. In selected cases, prior to the start of outpatient therapy, high-volume PD was used in a hospital setting until adequate metabolic and volume control was achieved, as previously published^31^.

In cases of contraindication to PD or refusal by family members or the patient, the initial method was HD. This involved the implantation of a specific double-lumen catheter and referral to start the outpatient method at the earliest opportunity, through four-hour sessions, three times a week, with adjustments according to the clinical and laboratory assessments made by the medical team.

### Patient Follow-Up

Data were collected directly from medical records. The first year of dialysis was selected as the observation period because of its higher morbidity and mortality rates^4^, its relatively understudied nature, and to provide a detailed analysis of different methods for initiating RRT. Over the twelve-month period, patients may have remained on the RRT method they first started, switched dialysis methods, recovered their kidney function, received a kidney transplant, or even evolved to death. Accurate data were available on the time spent in each state. This included information on more than one change (e.g. from HD to PD, and then transplantation). Information regarding dialysis access procedures, routine exams, dialysis therapy maintenance, hospitalizations, and the supply of medications related to end-stage CKD (ICD N18.0) from the Exceptional Circumstance Drug Dispensing Program (high-cost medications) was collected exclusively for the periods during which patients were undergoing dialysis methods, reflecting the costs associated with these.

### Calculation of Quality-Adjusted Life Year (QALY) in the First Year of Therapy

QALY is a standardized economic outcome that combines survival and quality of life. They are calculated by multiplying the time spent in a particular health state by the utility score (utility weight) associated with that state^32^. Utility estimates range from 0 to 1, with 0 representing death and 1 being perfect health. One year of life, from beginning to end, with a utility score of 1 (i.e., in perfect health), corresponds to one QALY.

The current working group has not applied utility scores. Thus, data imputation was based on the meta-analysis published by Wyld et al.^33^ It was assumed that every patient who experiences renal recovery would remain in a state similar to that of a pre-dialysis CKD patient, and that the utility score for transplantation would remain unchanged regardless of the time elapsed since surgery.

The area under the curve method was used to calculate QALYs^34^. The utility values were multiplied by the time spent in each state (dialysis, recovery of kidney function, transplant, death) during the first year after initiation of therapy. It was assumed that the patient would maintain the same utility during the entire period in each of the states.

### Obtaining Costs

Using a bottom-up or micro-costing methodology, data were collected during patient follow-up directly from their medical records. This provided real data on time in dialysis therapy, number of dialysis sessions performed, medications administered, dialysis access procedures performed, and events occurred. This enabled costs to be calculated for the funder based on the amounts reimbursed by the Unified Health System during the periods the patients were on dialysis throughout the follow-up period.

The cohort was analyzed by cost categories, namely direct medical costs related to medium- and high-complexity policies of the Unified Health System and related to dialysis treatment (routine exams, maintenance dialysis therapy, dialysis access, and medications from the Exceptional Circumstance Drug Dispensing Program in use related to ICD N18.0) as well as hospitalizations. Direct medical costs unrelated to SUS medium- and high-complexity policies (e.g. provision of medications to control blood pressure and diabetes mellitus), medications from the Exceptional Circumstance Drug Dispensing Program in use unrelated to ICD N18.0 (e.g. medications for refractory hypercholesterolemia, post-acute coronary syndrome, immunosuppressants) and during periods when the patient was not undergoing dialysis were not accounted for. Direct non-medical costs (e.g. provision of supplements and transportation) and indirect costs, including benefits and loss of working days (funded by Social Security), were also not used in the analysis. Costs associated with outpatient treatment of infections related to dialysis access were not included, as the SUS does not provide specific funding for these situations.

The costs for SUS associated with dialysis therapies, routine exams, and dialysis access were obtained by multiplying the number of procedures for each patient by the reimbursement value of the respective procedures recorded in the SUS Management System of the Table of Procedures, Medicines and Orthotics, Prostheses and Special Materials (OPME) (SIGTAP). The values available as of December 2019 were used.

Regarding hospitalizations, the cost to SUS was obtained by multiplying the number of days patients remained hospitalized during periods undergoing dialysis therapies by the value of code 03.05.01.017-4 - Treatment of intercurrences in chronic kidney disease patients under dialysis treatment (per day) from SIGTAP, available in December 2019.

To calculate the costs incurred by SUS in the supply of drugs related to end-stage CKD (ICD N18.0) under the Exceptional Circumstance Drug Dispensing Program (sevelamer, epoetin alfa, calcitriol, iron hydroxide, and cinacalcet hydrochloride), the values from closed bids available on the São Paulo State Electronic Purchasing Exchange (BEC/SP)35 until December 31, 2019, were used. If more than one value was available, the mean value for each medication was calculated. The values were as follows: sevelamer: R$ 3.05 per tablet; epoetin alfa 4,000 U: R$ 18.30/ampoule; epoetin alfa 10,000 U: R$ 48.65/ampoule; calcitriol 0.25 mcg: R$ 1.05/tablet; calcitriol 1 mcg: R$ 4.78/ampoule; iron hydroxide 100 mg: R$ 4.78/ampoule; cinacalcet hydrochloride 30 mg: R$ 3.72/tablet.

### Statistical Analysis

The study analysis was conducted on an intention-to-treat basis in order to assess the effects and costs, at the end of one year, of initiating unplanned dialysis therapy through either HD or PD. Based on the study protocol, data were entered into a Microsoft Excel 2013 spreadsheet. Typographical errors were then checked, and the analysis was performed using the SAS statistical program for Windows (version 9.2: SAS Institute, Cary, NC, USA, 2012).

Considering a type I error (alpha) of 5%, type II error (beta) of 20%, statistical power of the test at 80% and detection of a 15% difference in costs between groups, the calculated sample size for each group was 94 patients.

Descriptive analysis was performed for all patients included in the period, calculating measures of central tendency and dispersion for continuous variables and frequencies for categorical variables. When comparing groups, statistics were performed using the chi-square test for categorical variables and the T-test or Mann-Whitney test for continuous variables. Survival curves were created for patients who initiated RRT through both methods during the monitoring period, using Kaplan Meyer and log rank. The statistical difference was considered significant when p < 0.05.

### Economic Analysis

The study was a single study-based economic evaluation from the perspective of the payer, the Unified Health System, with a twelve-month follow-up period from the start of each patient’s outpatient RRT. The selected measures of effectiveness and utility were months of life in the first year and QALYs, respectively.

If there were differences in cost and effectiveness data, we proposed calculating the incremental cost-effectiveness ratio (ICER) and analyzing the data by bootstrapping, through data processing using Stata Statistical Software (Version 14, StataCorp, 2015), obtaining 2,000 resamples for each variable. This method allows for inferences about arithmetic means in asymmetric samples by assuming that the empirical distribution obtained is an adequate representation of the data distribution in the real population^34^. This allows for the calculation of ICER ranges, the development of acceptability curves, incremental cost-effectiveness plans, and the interpretation of results based on willingness-to-pay thresholds. It also enables univariate deterministic sensitivity analyses to be performed in order to assess parameter uncertainties (costs of hospitalizations, drugs, routine tests, and QALYs) and hypothetical scenarios of increased reimbursement for dialysis therapies and access to HD and PD, in accordance with the *Methodological Guidelines: Economic Evaluation Guidelines* from the Brazilian Ministry of Health^36^, with the construction of a tornado diagram using Microsoft Excel 2013. The chosen methodology more adequately replaces the construction of economic models as it is specific for primary data studies, according to guidelines on the subject^34,37^.

As the follow-up period for each patient is less than one year, the discount rate is not applicable.

### Ethical Aspects

The research was approved by the institution’s Research Ethics Committee (Opinion 1.951.845/2017, in Portuguese) and other required bodies based on Resolution 466/12 of the National Health Council. It was also registered at ClinicalTrial.gov (NCT02646436).

## Results

Two hundred and eight patients were included in the study, with nine excluded due to incomplete data and/or inconsistencies, and one due to a transfer from a dialysis center (data loss).

Regarding the composition of the groups, there were no differences between the group that started with HD and the one that started with PD in terms of age (58.65±15.57 *vs* 59.65±16.17 years, p = 0.658), male sex (44.4 *vs* 55.6%, p = 0.886), and prevalence of multiple comorbidities (81 *vs* 78%, p = 0.592). The groups also did not differ with regard to the cause of CKD (Table 1).

**Table 1:** Clinical characteristics of patients treated with unplanned dialysis

Clinical characteristics	Initiation via HD	Initiation via PD	P-value
	(n = 99)	(n = 99)	
Age (years)	58.65 ± 15.57	59.65 ± 16.17	0.658
Sex (male, n, %)	44 (44.4%)	55 (55.6%)	0.886
Two or more comorbidities (n, %)	81 (81.8%)	78 (78.8%)	0.592
CKD etiology (n, %)			0.674
Diabetes mellitus	30 (30.3%)	38 (38.4%)	0.295
Hypertension	19 (19.2%)	16 (16.2%)	0.71
Glomerulopathy	11 (11.1%)	11 (11.1%)	1
Obstructive	5 (5.1%)	7 (7.1%)	0.767
Other	34 (34.3%)	27 (27.3%)	0.356

Note: Values expressed as mean ± SD or number (percentage).

**Figure 1:**
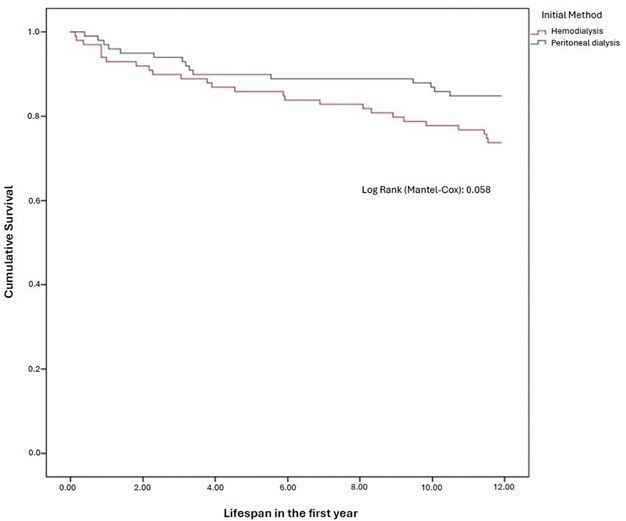
Kaplan-Meier curve for survival at 12 months from the start of outpatient dialysis.

Compared to the group that started on HD, the group that started on PD had a higher frequency of method transitions (1% *vs* 24.2%, p < 0.001), reflected in a lower percentage of patients who completed 12 months on the initial dialysis therapy (67.7% *vs* 52.5%, p = 0.029). There was no difference in the number of kidney transplants (4 *vs* 4%, p = 1.0) and the number of patients who experienced recovery of kidney function (2 *vs* 8, p = 0.052). Additionally, the groups were similar in the number of inpatient days [0 (0–9) *vs* 1 (0–8), p = 0.55] and infections [1 (0–1) *vs* 1 (0–2), p = 0.54]. However, the group that started with HD required more procedures related to dialysis access [3 (2–4) *vs* 1 (1–3), p < 0.001] during the period. Mortality (26% *vs* 15%, p = 0.054) and 12-month survival time [12 (11.51–12) *vs* 12 (12–12), p = 0.063] were similar between both groups. However, when calculating the number of QALYs obtained in the first year of therapy based on data from the meta-analysis by Wyld et al.^33^, it was observed that the group starting with PD had an advantage over the group starting with HD (0.59 ± 0.21 *vs* 0.65 ± 0.19; p = 0.035) (Table 2). The Kaplan-Meier curve for mortality is shown in Figure 1.

**Table 2:** Clinical outcomes

Outcomes	Initiation via HD	Initiation via PD	P-value
	(n = 99)	(n = 99)	
Survival time during follow-up (months)			
Median	12 (11.51–12)	12 (12–12)	0.063
Mean*	10.2 ± 3.65	10.84 ± 3.10	0.186
Total time on dialysis therapies during follow-up (months)	12 (11.9–12)	12 (8.32–12)	0.265
Maintenance of the dialysis method on which the patient started	67 (67.7%)	52 (52.5%)	0.029
Intermediate outcomes			
Method transition during follow-up	1 (1%)	24 (24.2%)	< 0.001
Recovery of kidney function during follow-up	2 (2%)	8 (8.1%)	0.052
Kidney transplantation during follow-up	4 (4%)	4 (4%)	1
Death after 12 months	26 (26.3%)	15 (15.2%)	0.054
Access procedures	3 (2–4)	1 (1–3)	< 0.001
Days in hospital	0 (0–9)	1 (0–8)	0.55
Episodes of dialysis-related infections	1 (0–1)	1 (0–2)	0.54
QALYs obtained in first year*	0.59 ± 0.21	0.65 ± 0.19	0.035

Note: Values expressed as mean ± SD, median (interquartile range) or number (percentage).

* Non-parametric data analyzed by parametric methods due to economic analysis.

In terms of costs, the average SUS expenditure on dialysis patients during the first year of therapy was similar in both groups (R$ 33,081.11 ± 12,760.52 *vs* R$ 35,478.96 ± 12,470.31, p = 0.607). The funding of dialysis procedures accounts for the largest share of the cost and is higher in the group that starts with PD (R$ 25,039.5 *vs* R$ 29,380.24, p = 0.002). The costs for medications from the Exceptional Circumstance Drug Dispensing Program are higher in the group starting with HD (R$ 5,187.78 *vs* R$ 4,103.37, p = 0.018), as are the expenses for dialysis access (R$ 1,713.04 *vs* R$ 976.57, p < 0.001), and routine exams (R$ 616.99 *vs* R$ 535.78, p = 0.01). As for hospitalization costs (R$ 523.78 *vs* R$ 482.99, p = 0.752), no differences were observed between the analyzed groups (Table 3).

**Table 3:** Cost analysis

Costs	Initiation via HD	Initiation via PD	P-value
	(n = 99)	(n = 99)	
Patient costs during dialysis therapies at follow-up	R$ 33,081.11 ± 12,760.52	R$ 35,478.96 ± 12,470.31	0.607
Costs by category			
Dialysis therapy*	R$ 25,039.5 (76%)	R$ 29,380.24 (83%)	0.002
Drugs	R$ 5,187.78 (16%)	R$ 4,103.37 (12%)	0.018
Hospitalizations*	R$ 523.78 (2%)	R$ 482.99 (1%)	0.752
Dialysis access*	R$ 1,713.04 (5%)	R$ 976.57 (3%)	< 0.001
Routine exams*	R$ 616.99 (2%)	R$ 535.78 (2%)	0.01
Cost per month of life in the first year*	R$ 3,462.89 ± 1,145.54	R$ 3,350.81 ± 759.03	0.418
Cost per QALY*	R$ 60,071.62 ± 19,700.96	R$ 56,090.17 ± 13,022.79	0.095

Note: Values expressed as mean ± SD or value (percentage of total costs).

* Non-parametric data analyzed by parametric methods due to economic analysis.

Thus, initiating with either PD or HD does not differ in terms of costs and effectiveness (months of life in the first year), although there may be better utility in the group that starts with PD. No differences were observed when analyzing the costs related to dialysis patients per month of life in the first year (R$ 3,462.89 ± 1,145.54 *vs* R$ 3,350.81 ± 759.03, p = 0.418) and the costs related to dialysis patients per QALY obtained in the first year (R$ 60,071.62 ± 19,700.96 *vs* R$ 56,090.17 ± 13,022.79, p = 0.095). Due to the absence of differences in costs and effectiveness, the therapies are considered equivalent, and ICER calculations and detailed analyses are deemed useless (Table 3). The group decided to interpret the utility results with caution and decided not to proceed with further analyses due to data extrapolated from a study of a different population, as detailed in the discussion session.

## Discussion

When discussing the reality of advanced-stage CKD, two prominent issues in developing countries are the lack of access to renal replacement therapies^38,39^ and the initiation of dialysis therapy in an unplanned manner^5,6,7,8,9,40,41^. In a scenario with limited resources, costs directly impact the morbidity and mortality of patients requiring such treatment^4,42^. PD is a therapeutic option with the potential to alter this reality; however, it remains underutilized^6,10,11^ due to factors ranging from lack of technical knowledge to outdated concepts based on old studies that cast doubt on the results of PD.

The study revealed that, in the context of unplanned RRT, starting with peritoneal dialysis was equivalent to starting with HD, with comparable costs and effectiveness. The result of obtaining higher QALYs in the first year when starting with PD, inferring better utility, while positive and encouraging, was not given enough weight to be included in a detailed economic analysis due to being the variable with the greatest bias in the study. The utility data were derived from an international meta-analysis^33^, rather than from the state or Brazilian populations.

It is also worth noting that, in addition to the clinical advantages already mentioned, PD presents relevant political and economic factors not addressed in this study. By opting for a policy that promotes PD, there are notable savings on infrastructure costs. Since this therapy is performed at the patient’s home rather than within a dialysis center, it does not require the construction or adaptation of large building structures or the acquisition of specific high-cost machinery. Regarding facilities, according to Collegiate Board Resolution (RDC) 50 of 2002^30^, a unit exclusively for peritoneal dialysis may be accredited and initiated in health units that comply with the RDC, whether public or private, hospital or outpatient. There is often no need for material acquisition or even renovations, provided there are available rooms that meet the minimal requirements. For example, this could easily be implemented in private clinics with vacant offices or even in primary healthcare units with idle rooms. For the establishment of a hemodialysis center, minimally, considering a unit that does not reprocess capillaries and has no room for hepatitis B patients , in addition to a large hall or multiple small rooms for the hemodialysis chairs and/or beds, it would be necessary to have a treatment area and a reservoir for treated dialysis water, an area for fistula washing, and adjustments proportional to the number of chairs and/or beds, at least in relation to the nursing and services station and the patient recovery room. Regarding equipment, the commonly adopted practice by suppliers of PD materials is a lending arrangement, where the APD machine is provided at no cost to the executing unit, including maintenance^43^. Although hemodialysis machines could also be provided on a loan or rental basis, thus diluting the initial costs, it is necessary to purchase chairs and/or beds and a central water treatment and distribution system, which in 2022 was worth more than 260,000 reais^44,45^.

The need for human, energy and water resources is also lower, as are regulations, licenses and quality controls^46,47^. Furthermore, from a logistical perspective, there is no need for patients to be transported, typically three times a week, to attend the sessions. These characteristics are particularly important in developing countries when evaluating the expansion of dialysis slots. In Brazil, this chronic issue occasionally receives media attention, particularly when outpatient HD slots are unavailable. In such cases, patients who start their RRT with HD do so during hospitalization remain in hospital beds until a slot becomes available, which can take weeks or even months^23,24,25^.

The study’s strengths include the use of primary data, reducing uncertainties, inferences, and, consequently, the need for corrections. Information extracted directly from patients’ electronic medical records provides solidity and reliability to the data across all periods, including during method changes. The pragmatic nature of the study, with real-world data, allows for easier external validation, despite the primary analysis and the fact that it is a single-center study. The choice of an intention-to-treat approach makes both clinical and economic evaluation more fluid and conclusive. It is not restricted to comparing two dialysis methods, but rather assesses the strategy of starting with one method or the other; a public health policy is evaluated.

However, there are limitations. Initially, it is a single-center study. As an example, it is worth noting that the studied population has a prevalence of double-lumen catheter use as a dialysis access for hemodialysis greater than 60%^19,48^. These figures are significantly higher than the 2022 Brazilian Dialysis Survey data, which reported a prevalence of 20.9% of long-term accesses and 8.3% of short-term accesses among HD patients^10^. This characteristic may explain the greater number of interventions in dialysis access and may have interfered with mortality in the HD group, which reached levels close to statistical significance. Another factor would be the limited follow-up time. The group’s decision not to build economic models in order to extend it is justified so as to detail the events and costs incurred in the year of access to unplanned RRT, which has higher morbidity and mortality, thus allowing for public health policies to be discussed. Randomization was not performed due to the aforementioned difficulties, which could result in biases, particularly selection bias, with patients in better health being referred to the peritoneal dialysis group, despite the absence of differences between the studied groups. In addition, the analysis and accounting of events and costs were conducted only during the periods in which the patient was undergoing dialysis, excluding data from other periods, except for the time factor. The perspective of the study was that of SUS at the federal level through the reimbursements at the time of the study. This does not reflect the actual costs of providing units and is not necessarily proportional to current reimbursements. There are also the previously mentioned criticisms regarding the utility scores used.

The follow-up period and the choice of intention-to-treat analysis hinder comparisons with other economic analyses of unplanned dialysis, such as that by Liu et al.^29^, which reported lower costs, perhaps because it overlooked the high percentage of method transitions to hemodialysis over the months, figures that were included in this study.

Proposals such as unplanned PD programs have been proven effective^5,19,49,50,51^, but increasing the use of the method involves economic, political and cultural issues. In the case of the HC-FMB Dialysis Unit experience, the growth of the unplanned PD program allowed a large number of patients to access outpatient treatment, thus avoiding hospitalizations while waiting for outpatient dialysis slots and possibly preventing deaths before reaching the referral service.

## Conclusion

The results show that starting RRT with PD is a comparable option to starting RRT with HD in patients requiring unplanned methods, with similar outcomes and costs for maintaining patients on RRT. However, the low initial investment in infrastructure and the machinery required to establish PD slots strengthen its position as a public health policy for the expansion of RRT in developing countries. Studies with primary data on quality of life are needed to better assess the superiority in QALYs of initiating RRT in PD compared to HD, and consequently their cost-utility.

## Supplementary Material

The following online material is available for this article:

Table s1 - SUS funding for the maintenance of dialysis-based renal replacement therapies

Table s2 - Testing protocol according to the Ministry of Health

Table s3 - SUS funding for protocol exams

Table s4 - SUS funding for the creation and intervention of dialysis accesses

Table s5 - Medication pricing according to the São Paulo State Purchasing Exchange

## References

[1] Summary of Recommendation Statements (2013). KDIGO 2012 clinical practice guideline for the evaluation and management of chronic kidney disease. Kidney Int Suppl.

[2] Brasil, Ministério da Saúde (2014). Diretrizes clínicas para o cuidado ao paciente com doença renal crônica no Sistema Único de Saúde [Internet]. http://bvsms.saude.gov.br/bvs/publicacoes/diretrizes_clinicas_cuidado_paciente_renal.pdf.

[3] Levin A, Ahmed SB, Carrero JJ, Foster B, Francis A, Hall RK (2024). Executive summary of the KDIGO 2024 clinical practice guideline for the evaluation and management of chronic kidney disease: known knowns and known unknowns. Kidney Int.

[4] U.S. Department of Health and Human Services (2023). Unites States renal data system annual data report 2023 [Internet]. https://usrds-adr.niddk.nih.gov/.

[5] Dias DB, Banin V, Mendes ML, Barretti P, Ponce D (2016). Peritoneal dialysis as an option for unplanned initiation of chronic dialysis. Hemodial Int.

[6] Thomé FS, Sesso RC, Lopes AA, Lugon JR, Martins CT (2019). Brazilian chronic dialysis survey 2017. J Bras Nefrol.

[7] Perl J, Wald R, McFarlane P, Bargman JM, Vonesh E, Na Y (2011). Hemodialysis vascular access modifies the association between dialysis modality and survival. J Am Soc Nephrol.

[8] Heaf JG, Løkkegaard H, Madsen M (2002). Initial survival advantage of peritoneal dialysis relative to haemodialysis. Nephrol Dial Transplant.

[9] Termorshuizen F, Korevaar JC, Dekker FW, Van Manen JG, Boeschoten EW, Krediet RT (2003). Hemodialysis and peritoneal dialysis: comparison of adjusted mortality rates according to the duration of dialysis: analysis of The Netherlands Cooperative Study on the Adequacy of Dialysis 2. J Am Soc Nephrol.

[10] Nerbass FB, Lima HN, Moura JA, Lugon JR, Sesso R (2023). Censo brasileiro de diálise 2022. Braz J Nephrol.

[11] Neves PDMM, Sesso RCC, Thomé FS, Lugon JR, Nasicmento MM (2020). Brazilian dialysis census: analysis of data from the 2009-2018 decade. J Bras Nefrol.

[12] Lok CE, Huber TS, Lee T, Shenoy S, Yevzlin AS, Abreo K (2020). KDOQI clinical practice guideline for vascular access: 2019 update. Am J Kidney Dis.

[13] Blake PG, Jain AK (2018). Urgent start peritoneal dialysis: defining what it is and why it matters. Clin J Am Soc Nephrol.

[14] Korevaar JC, Feith GW, Dekker FW, van Manen JG, Boeschoten EW, Bossuyt PMM (2003). Effect of starting with hemodialysis compared with peritoneal dialysis in patients new on dialysis treatment: a randomized controlled trial. Kidney Int.

[15] Vonesh EF, Snyder JJ, Foley RN, Collins AJ (2006). Mortality studies comparing peritoneal dialysis and hemodialysis: what do they tell us?. Kidney Int Suppl.

[16] Zang XJ, Yang B, Du X, Mei CL (2019). Urgent-start peritoneal dialysis and patient outcomes: a systematic review and meta-analysis. Eur Rev Med Pharmacol Sci.

[17] Ding X, Gao W, Guo Y, Cai Q, Bai Y (2022). Comparison of mortality and complications between urgent-start peritoneal dialysis and urgent-start hemodialysis: a systematic review and meta-analysis. Semin Dial.

[18] Ivarsen P, Povlsen JV (2014). Can peritoneal dialysis be applied for unplanned initiation of chronic dialysis?. Nephrol Dial Transplant.

[19] Dias DB, Mendes ML, Caramori JT, Falbo dos Reis P, Ponce D (2021). Urgent-start dialysis: comparison of complications and outcomes between peritoneal dialysis and haemodialysis. Perit Dial Int.

[20] Koch M, Kohnle M, Trapp R, Haastert B, Rump LC, Aker S (2012). Comparable outcome of acute unplanned peritoneal dialysis and haemodialysis. Nephrol Dial Transplant.

[21] Lobbedez T, Lecouf A, Ficheux M, Henri P, Hurault de Ligny B, Ryckelynck JP (2008). Is rapid initiation of peritoneal dialysis feasible in unplanned dialysis patients? A single-centre experience. Nephrol Dial Transplant.

[22] Alkatheeri AMA, Blake PG, Gray D, Jain AK (2016). Success of urgent-start peritoneal dialysis in a large Canadian renal program. Perit Dial Int.

[23] JCNET (2024). Pacientes precisam esperar até 150 dias internados para passar por hemodiálise [Internet]. https://sampi.net.br/bauru/noticias/2820874/politica/2024/03/pacientes-precisam-esperar-ate-150-dias-internados-para-passar-por-hemodialise-.

[24] Associação Brasileira dos Centros de Diálise e Transplante (2017). Sem vagas em clínicas, pacientes renais ficam internados em hospitais à espera de hemodiálise [Internet]. https://www.abcdt.org.br/2017/10/sem-vagas-em-clinicas-pacientes-renais-ficam-internados-em-hospitais-espera-de-hemodialise/.

[25] G1 (2024). Falta de vaga para hemodiálise no SUS faz paciente ficar internado por meses na região [Internet]. https://g1.globo.com/sp/campinas-regiao/noticia/2024/03/09/falta-de-vaga-para-hemodialise-no-sus-faz-paciente-ficar-internado-por-meses-na-regiao.ghtml.

[26] Klarenbach SW, Tonelli M, Chui B, Manns BJ (2014). Economic evaluation of dialysis therapies. Nat Rev Nephrol.

[27] Chang YT, Hwang JS, Hung SY, Tsai MS, Wu JL, Sung JM (2016). Cost-effectiveness of hemodialysis and peritoneal dialysis: a national cohort study with 14 years follow-up and matched for comorbidities and propensity score. Sci Rep.

[28] Atapour A, Eshaghian A, Taheri D, Dolatkhah S (2015). Hemodialysis versus peritoneal dialysis, which is cost-effective?. Saudi J Kidney Dis Transpl.

[29] Liu FX, Ghaffari A, Dhatt H, Kumar V, Balsera C, Wallace E (2014). Economic evaluation of urgent-start peritoneal dialysis versus urgent-start hemodialysis in the United States. Medicine.

[30] Brasil, Ministério da Saúde (2002). Resolução da Diretoria Colegiada (RDC) nº 50, de 21 de fevereiro de 2002 [Internet]. https://bvsms.saude.gov.br/bvs/saudelegis/anvisa/2002/rdc0050_21_02_2002.html.

[31] Gabriel DP, Caramori JT, Martim LC, Barretti P, Balbi AL (2008). High volume peritoneal dialysis vs daily hemodialysis: a randomized, controlled trial in patients with acute kidney injury. Kidney Int Suppl.

[32] Whitehead SJ, Ali S (2010). Health outcomes in economic evaluation: the QALY and utilities. Br Med Bull.

[33] Wyld M, Morton RL, Hayen A, Howard K, Webster AC (2012). A systematic review and meta-analysis of utility-based quality of life in chronic kidney disease treatments. PLoS Med.

[34] Glick H, Doshi JA, Sonnad SS, Polsky D (2015). Economic evaluation in clinical trials.

[35] Bolsa Eletrônica de Compras – BECSP [Internet] (2021). https://www.bec.sp.gov.br/BECSP/Home/Home.aspx.

[36] Brasil, Ministério da Saúde (2014). Diretrizes metodológicas: diretriz de avaliação econômica.

[37] Ramsey SD, Willke RJ, Glick H, Reed SD, Augustovski F, Jonsson B (2015). Cost-effectiveness analysis alongside clinical trials II-An ISPOR Good Research Practices Task Force report. Value Health.

[38] Couser WG, Remuzzi G, Mendis S, Tonelli M (2011). The contribution of chronic kidney disease to the global burden of major noncommunicable diseases. Kidney Int.

[39] Eggers P (2011). Has the incidence of end-stage renal disease in the USA and other countries stabilized? Current opinion in nephrology and hypertension. Curr Opin Nephrol Hypertens.

[40] Silva TNV, de Marchi D, Mendes ML, Barretti P, Ponce D (2014). Approach to prophylactic measures for central venous catheter-related infections in hemodialysis: a critical review. Hemodial Int.

[41] Mendes ML, Castro JH, Silva TN, Barretti P, Ponce D (2014). Effective use of alteplase for occluded tunneled venous catheter in hemodialysis patients. Artif Organs.

[42] National Kidney Foundation (2020). Global facts: about kidney disease [Internet]. https://www.kidney.org/kidneydisease/global-facts-about-kidney-disease.

[43] São Paulo (2023). Edital de pregão eletrônico do Hospital do servidor público municipal de Secretaria Municipal da Saúde da Prefeitura da cidade de São Paulo [Internet]. https://www.prefeitura.sp.gov.br/cidade/secretarias/upload/saude/hospital_do_servidor_publico_municipal/acesso_a_informacao/2023/020_2023.pdf.

[44] Associação Brasileira dos Centros de Diálise e Transplante (2021). Planilha de custos [Internet]. https://www.abcdt.org.br/planilha-de-custos/.

[45] Souza WGD (2022). Água potável: HUCF investe em ETA, análise da qualidade e uso em Diálise [Internet]. https://unimontes.br/agua-potavel-hucf-investe-em-eta-analise-da-qualidade-e-uso-em-dialise/.

[46] Brasil, Ministério da Saúde (2018). Portaria nº 1.675, de 7 de junho de 2018 [Internet]. Diário Oficial da União.

[47] Rocha Fo JA (2024). O olhar da Vigilância Sanitária e os serviços de hemodiálise [Internet]. https://www.prefeitura.sp.gov.br/cidade/secretarias/upload/saude/apresentacao_Jose_Alves_Rocha_Filho.pdf.

[48] Bitencourt Dias D, Mendes ML, Burgugi Banin V, Barretti P, Ponce D (2017). Urgent-start peritoneal dialysis: the first Year of Brazilian experience. Blood Purif.

[49] Dias DB, Banin V, Mendes ML, Barretti P, Ponce D (2016). Peritoneal dialysis can be an option for unplanned chronic dialysis: initial results from a developing country. Int Urol Nephrol.

[50] Mendes ML, Alves CA, Bucuvic EM, Dias DB, Ponce D (2017). Peritoneal dialysis as the first dialysis treatment option initially unplanned. J Bras Nefrol.

[51] Dias DB, Mendes ML, Alves CA, Caramori JT, Ponce D (2020). Peritoneal dialysis as an urgent-start option for incident patients on chronic renal replacement therapy: world experience and review of literature. Blood Purif.

