# Supplementary material for: Economic analysis of hemodialysis and urgent-start peritoneal dialysis therapies
Source: J Bras Nefrol. 2025 Jan 10;47(1):e20240051. doi: 10.1590/2175-8239-JBN-2024-0051en (PMC11723605; doi:10.1590/2175-8239-JBN-2024-0051en)
Supplement: Supplementary file 1 [file 2175-8239-jbn-47-1-e20240051-suppl1.pdf]

Braz. J. Nephrol.
https://doi.org/10.1590/2175-8239-JBN-2024-0051pt

Material Suplementar para “Análise econômica das terapias hemodiálise e diálise peritoneal de início urgente”

Tabela s1 - Repasse do SUS para a manutenção de terapias renais substitutivas dialíticas.

Procedimentos dialíticos e materiais para faturamento	Código do item	Valor do repasse	Faturamento
Hemodiálise (máximo 3 sessões por semana)	03.05.01.010-7	R$ 194,20	Por sessão
Hemodiálise (máximo 1 sessão por semana – excepcionalidade)	03.05.01.009-3	R$ 194,20	Por sessão
Diálise peritoneal intermitente DPI (máximo 2 sessões por semana)	03.05.01.002-6	R$ 121,51	Por sessão
Diálise peritoneal intermitente DPI (1 sessão por semana – excepcionalidade)	03.05.01.001-8	R$ 121,74	Por sessão
Treinamento de paciente submetido a diálise peritoneal – DPAC-DPA (9 dias)	03.05.01.018-2	R$ 55,13	Por treinamento
Conjunto de troca para treinamento de paciente submetido a DPA/DPAC (9 dias) correspondente a 36 unidades	07.02.10.007-2	R$ 609,39	Por treinamento
Manutenção e acompanhamento domiciliar de paciente submetido a DPA/DPAC	03.05.01.016-6	R$ 358,06	Mensal
Conjunto de troca para DPA (paciente/mês com instalação domiciliar e manutenção da máquina cicladora)	07.02.10.004-8	R$ 2.511,49	Mensal
Conjunto de troca para paciente submetido a DPAC (paciente/mês) correspondente a 120 unidades	07.02.10.006-4	R$ 1.893,68	Mensal
Conjuntos de troca para paciente submetido a DPAC (paciente/15 dias)	07.02.10.008-0	R$ 946,84	Mensal
Conjunto de troca para paciente submetido a DPA (paciente/15 dias com instalação domiciliar e manutenção de máquina cicladora)	07.02.10.005-6	R$ 1.255,74	Mensal

Nota: Texto extraído diretamente do site do SIGTAP. Subitens são opções de procedimentos geralmente faturados em conjunto com o item imediatamente acima.
