# Supplementary material for: Economic analysis of hemodialysis and urgent-start peritoneal dialysis therapies
Source: J Bras Nefrol. 2025 Jan 10;47(1):e20240051. doi: 10.1590/2175-8239-JBN-2024-0051en (PMC11723605; doi:10.1590/2175-8239-JBN-2024-0051en)
Supplement: Supplementary file 2 [file 2175-8239-jbn-47-1-e20240051-suppl2.pdf]

Braz. J. Nephrol.
https://doi.org/10.1590/2175-8239-JBN-2024-0051pt

Material Suplementar para "Análise econômica das terapias hemodiálise e diálise peritoneal de início urgente"

Tabela s2 - Protocolo de exames segundo o Ministério da Saúde.

Tipo de exame	Periodicidade	
	Hemodiálise	Diálise peritoneal
Dosagem de cálcio	12 vezes ao ano	12 vezes ao ano
Dosagem de fósforo	12 vezes ao ano	12 vezes ao ano
Dosagem de potássio	12 vezes ao ano	12 vezes ao ano
Dosagem de sódio	12 vezes ao ano	12 vezes ao ano
Dosagem de transaminase glutâmico-pirúvica (TGP)	12 vezes ao ano	Fora do protocolo
Dosagem de ureia	12 vezes ao ano	Fora do protocolo
Dosagem de ureia (pós-sessão de hemodiálise)	12 vezes ao ano	Fora do protocolo
Dosagem de creatinina	12 vezes ao ano	12 vezes ao ano
Dosagem de glicose*	12 vezes ao ano	12 vezes ao ano
Dosagem de hemoglobina	8 vezes ao ano	8 vezes ao ano
Hematócrito	8 vezes ao ano	8 vezes ao ano
Hemograma completo	4 vezes ao ano	4 vezes ao ano
Dosagem de proteínas totais e frações	4 vezes ao ano	4 vezes ao ano
Dosagem de paratormônio	4 vezes ao ano	4 vezes ao ano
Dosagem de fosfatase alcalina	4 vezes ao ano	4 vezes ao ano
Dosagem de ferritina	4 vezes ao ano	4 vezes ao ano
Dosagem de ferro sérico	4 vezes ao ano	4 vezes ao ano
Dosagem de transferrina	4 vezes ao ano	4 vezes ao ano
Dosagem de hemoglobina glicosilada**	4 vezes ao ano	4 vezes ao ano
Pesquisa de anticorpos contra antígeno de superfície do vírus da hepatite B (anti-HBS)	2 vezes ao ano	Fora do protocolo
Dosagem de 25 hidroxivitamina D	2 vezes ao ano	2 vezes ao ano
Pesquisa de antígeno de superfície do vírus da hepatite B (HBSAG)	2 vezes ao ano	Fora do protocolo
Pesquisa de anticorpos contra o vírus da hepatite C (anti-HCV)	2 vezes ao ano	Fora do protocolo

Tipo de exame	Periodicidade	
	Hemodiálise	Diálise peritoneal
Dosagem de colesterol total	1 vez ao ano	2 vezes ao ano
Dosagem de colesterol HDL	1 vez ao ano	2 vezes ao ano
Dosagem de triglicerídeos	1 vez ao ano	2 vezes ao ano
Dosagem de alumínio	1 vez ao ano	1 vez ao ano
Dosagem de hormônio tireoestimulante (TSH)	1 vez ao ano	1 vez ao ano
Dosagem de tiroxina livre (T4 livre)	1 vez ao ano	1 vez ao ano
Pesquisa de anticorpos anti-HIV-1 + HIV-2 (ELISA)	1 vez ao ano	Fora do protocolo
Radiografia de tórax (PA e Perfil)	1 vez ao ano	1 vez ao ano
Eletrocardiograma	1 vez ao ano	1 vez ao ano
Clearance de creatinina	Fora do protocolo	2 vezes ao ano
Clearance de ureia	Fora do protocolo	2 vezes ao ano
Adequação em diálise peritoneal (5 dosagens de ureia)	Fora do protocolo	2 vezes ao ano
Valor estimado de repasse em 12 meses	R$ 752,39	R$ 606,75
Valor médio de repasse por mês	R$ 62,70	R$ 50,56

* Glicose é realizada (e faturada) apenas nos pacientes portadores de diabetes *mellitus* em hemodiálise (aproximadamente 50% dos pacientes), enquanto na diálise peritoneal é realizada em 100% dos pacientes.

** Hemoglobina glicosilada é realizada (e faturada) apenas nos pacientes portadores de diabetes *mellitus* em hemodiálise e diálise peritoneal (aproximadamente 50% dos pacientes).
