# Supplementary material for: Economic analysis of hemodialysis and urgent-start peritoneal dialysis therapies
Source: J Bras Nefrol. 2025 Jan 10;47(1):e20240051. doi: 10.1590/2175-8239-JBN-2024-0051en (PMC11723605; doi:10.1590/2175-8239-JBN-2024-0051en)
Supplement: Supplementary file 3 [file 2175-8239-jbn-47-1-e20240051-suppl3.pdf]

Braz. J. Nephrol.
https://doi.org/10.1590/2175-8239-JBN-2024-0051pt

Material Suplementar para "Análise econômica das terapias hemodiálise e diálise peritoneal de início urgente"

Tabela s3 - Repasse do SUS para exames protocolares.

Exames para faturamento	Código do item	Valor do repasse
Hemograma completo	02.02.02.038-0	R$ 4,11
Dosagem de hemoglobina	02.02.02.030-4	R$ 1,53
Hematócrito	02.02.02.037-1	R$ 1,53
Dosagem de cálcio	02.02.01.021-0	R$ 1,85
Dosagem de creatinina	02.02.01.031-7	R$ 1,85
Dosagem de fósforo	02.02.01.043-0	R$ 1,85
Dosagem de glicose	02.02.01.047-3	R$ 1,85
Dosagem de potássio	02.02.01.060-0	R$ 1,85
Dosagem de sódio	02.02.01.063-5	R$ 1,85
Dosagem de transaminase glutâmico-pirúvica (TGP)	02.02.01.065-1	R$ 2,01
Dosagem de ureia	02.02.01.069-4	R$ 1,85
Dosagem de colesterol HDL	02.02.01.027-9	R$ 3,51
Dosagem de colesterol total	02.02.01.029-5	R$ 1,85
Dosagem de ferritina	02.02.01.038-4	R$ 15,59
Dosagem de ferro sérico	02.02.01.039-2	R$ 3,51
Dosagem de fosfatase alcalina	02.02.01.042-2	R$ 2,01
Dosagem de hemoglobina glicosilada	02.02.01.050-3	R$ 7,86
Dosagem de proteínas totais e frações	02.02.01.062-7	R$ 1,85
Dosagem de transferrina	02.02.01.066-0	R$ 4,12
Dosagem de triglicerídeos	02.02.01.067-8	R$ 3,51
Dosagem de 25 hidroxivitamina D	02.02.01.076-7	R$ 15,24
Dosagem de paratormônio	02.02.06.027-6	R$ 43,13
Dosagem de tiroxina livre (T4 livre)	02.02.06.038-1	R$ 11,60
Dosagem de alumínio	02.02.07.008-5	R$ 27,50

Exames para faturamento	Código do item	Valor do repasse
Dosagem de hormônio tireoestimulante (TSH)	02.02.06.025-0	R$ 8,96
Clearance de creatinina	02.02.05.002-5	R$ 3,51
Clearance de ureia	02.02.05.004-1	R$ 3,51
Pesquisa de anticorpos anti-HIV-1 + HIV-2 (ELISA)	02.02.03.030-0	R$ 10,00
Pesquisa de anticorpos contra o vírus da hepatite C (anti-HCV)	02.02.03.067-9	R$ 18,55
Pesquisa de antígeno de superfície do vírus da hepatite B (HBSAG)	02.02.03.097-0	R$ 18,55
Pesquisa de anticorpos contra antígeno de superfície do vírus da hepatite B (anti-HBS)	02.02.03.063-6	R$ 18,55
Eletrocardiograma	02.11.02.003-6	R$ 5,15
Radiografia de tórax (PA e Perfil)	02.04.03.017-0	R$ 9,50

Nota: Texto extraído diretamente do site do SIGTAP.O faturamento dos itens acima é por exame.
