# Supplementary material for: Economic analysis of hemodialysis and urgent-start peritoneal dialysis therapies
Source: J Bras Nefrol. 2025 Jan 10;47(1):e20240051. doi: 10.1590/2175-8239-JBN-2024-0051en (PMC11723605; doi:10.1590/2175-8239-JBN-2024-0051en)
Supplement: Supplementary file 4 [file 2175-8239-jbn-47-1-e20240051-suppl4.pdf]

Braz. J. Nephrol. (J. Bras. Nefrol.)
https://doi.org/10.1590/2175-8239-JBN-2024-0051pt

Material Suplementar para "Análise econômica das terapias hemodiálise e diálise peritoneal de início urgente"

Tabela s4 - Repasse do SUS para criação e intervenção em acessos dialíticos.

Procedimentos e itens para faturamento	Código do item	Valor do repasse	Forma de faturamento
Implante de cateter duplo lúmen para hemodiálise	04.18.01.006-4	R$ 115,81	Por procedimento
Cateter para subclávia duplo lúmen para hemodiálise	07.02.10.002-1	R$ 64,76	Por procedimento
Dilatador para implante de cateter duplo lúmen	07.02.10.009-9	R$ 21,59	Por procedimento
Guia metálico para introdução de cateter duplo lúmen	07.02.10.010-2	R$ 15,41	Por procedimento
Implante de cateter de longa permanência para hemodiálise	04.18.01.004-8	R$ 200,00	Por procedimento
Cateter de longa permanência para hemodiálise	07.02.10.001-3	R$ 482,34	Por procedimento
Confecção de fístula arteriovenosa para acesso	04.06.02.008-6	R$ 600,00	Por procedimento
Confecção de fístula arteriovenosa com enxertia de politetrafluoretileno (PTFE)	04.18.01.001-3	R$ 1.453,85	Por procedimento
Intervenção em fístula arteriovenosa	04.18.02.001-9	R$ 600,00	Por procedimento
Ligadura de fístula arteriovenosa	04.18.02.002-7	R$ 600,00	Por procedimento
Implante de cateter tipo Tenckhoff ou similar para DPA/DPAC	04.18.01.008-0	R$ 400,00	Por procedimento
Cateter tipo Tenckhoff/similar de longa permanência para DPI/DPAC/DPA	07.02.10.003-0	R$ 149,75	Por procedimento
Retirada de cateter tipo Tenckhoff / similar de longa permanência*	04.18.02.003-5	R$ 400,00	Por procedimento

Nota: Texto extraído diretamente do site do SIGTAP. Subitens são relacionados a procedimentos geralmente faturados em conjunto com o item imediatamente acima.
*Procedimento também utilizado para retirada de cateter de longa permanência para hemodiálise
