# Supplementary material for: Economic analysis of hemodialysis and urgent-start peritoneal dialysis therapies
Source: J Bras Nefrol. 2025 Jan 10;47(1):e20240051. doi: 10.1590/2175-8239-JBN-2024-0051en (PMC11723605; doi:10.1590/2175-8239-JBN-2024-0051en)
Supplement: Supplementary file 5 [file 2175-8239-jbn-47-1-e20240051-suppl5.pdf]

Braz. J. Nephrol. (J. Bras. Nefrol.)

https://doi.org/10.1590/2175-8239-JBN-2024-0051pt

Material Suplementar para "Análise econômica das terapias hemodiálise e diálise peritoneal de início urgente"

Tabela s5 - Valores das medicações segundo a Bolsa de Compras do Estado de São Paulo.

Medicação	Código do item	Meses avaliados e valores obtidos												Valor considerado
		mar/16	ago/16	dez/16	mar/17	mar/18	maio/18	set/18	out/18	maio/19	jun/19	jul/19	set/19	
Alfaepoetina 4.000 U (amp)	106054	R$ 18,30	-	-	-	-	-	-	-	-	-	-	-	R$ 18,30
Alfaepoetina 10.000 U (amp)	106046	-	-	-	R$ 55,00	R$ 42,89	-	-	R$ 40,70	R$ 56,00	-	-	-	R$ 48,65
Hidróxido de ferro 100 mg (amp)	2753219	-	-	-	-	-	-	-	-	R$ 4,11	R$ 5,45	-	-	R$ 4,78
Calcitriol 0,25 mcg (comp)	108138	-	-	-	-	-	R$ 0,61	-	-	-	-	R$ 1,49	-	R$ 1,05
Calcitriol 1 mcg (amp)	108111	-	R$ 25,30	-	-	-	-	-	-	-	-	-	-	R$ 25,30
Cinacalcete 30 mg (comp)	3521567	-	-	-	-	-	-	R$ 3,67	-	-	-	-	R$ 3,76	R$ 3,72
Sevelamer 800 mg (comp)	1466160	-	-	R$ 3,05	-	-	-	-	-	-	-	-	-	R$ 3,05

Nota: O valor considerado, em caso de mais de uma cotação, resulta da média dos valores encontrados.
