# Supplementary material for: Economic analysis of hemodialysis and urgent-start peritoneal dialysis therapies
Source: J Bras Nefrol. 2025 Jan 10;47(1):e20240051. doi: 10.1590/2175-8239-JBN-2024-0051en (PMC11723605; doi:10.1590/2175-8239-JBN-2024-0051en)
Supplement: Translation

# Translation: Análise econômica das terapias hemodiálise e diálise peritoneal de início urgente

**DOI:** 10.1590/2175-8239-JBN-2024-0051pt

**Authors:** Alexandre Minetto Brabo, Dayana Bitencourt Dias, Everton Nunes da Silva, Daniela Ponce

**Affiliations:** 1 Universidade Estadual Paulista, Faculdade de Medicina, Departamento de Clínica Médica, Botucatu, SP, Brasil.; 2 Universidade de Brasília, Departamento de Saúde Coletiva, Brasília, DF, Brasil.

**Keywords:** Diálise Peritoneal, Hemodiálise, Avaliação Econômica em Saúde, Avaliação de Custo-Efetividade, Análise Custo-Eficiência, Política de Saúde, Política Informada por Evidências

## Abstract

**Introdução:**

O início da terapia renal substitutiva (TRS) de modo não planejado em portadores de doença renal crônica (DRC) é situação frequente mundialmente. Nesse cenário, a diálise peritoneal (DP) tem se evidenciado como opção terapêutica em comparação à hemodiálise (HD). Na TRS planejada, os custos da DP são inferiores aos da HD, porém a literatura carece dessas análises quando o início é urgente.

**Objetivo:**

Avaliar clínica e economicamente, na perspectiva do Sistema Único de Saúde (SUS), a estratégia de iniciar TRS não planejada por HD e DP em pacientes durante seu primeiro ano de terapia.

**Metodologia:**

Estudo quase experimental com análise de custo-efetividade a partir de dados primários de pacientes incidentes em TRS, em horizonte temporal de doze meses, por intenção de tratamento. A coleta de dados ocorreu de maneira prospectiva, diretamente dos prontuários médicos, computando dados de utilização de terapia dialítica, medicações de alto custo, procedimentos em acessos dialíticos e eventos ocorridos, com estimativa de custos pelos valores repassados pelo SUS. Na análise econômica, foi proposta a aplicação de método de *bootstrapping* e construção de gráficos.

**Resultados:**

No fim de um ano, não houve diferenças entre custos e efetividade ao iniciar a TRS de maneira não planejada por DP e HD.

**Conclusão:**

Iniciar TRS por DP é opção semelhante a iniciar por HD em pacientes com necessidade de métodos não planejados. A mínima necessidade de investimento inicial para criação de vagas de DP é fator que a fortalece como política de saúde pública para expansão da TRS em países em desenvolvimento.

## Introdução

A doença renal crônica (DRC) é um estado de saúde complexo associado a morbidade e mortalidade aumentada e alta demanda por recursos dos sistemas de saúde^1,2,3,4^. Considerando a eficiência limitada das medidas de controle de progressão da DRC, parte dos pacientes terá necessidade de terapia renal substitutiva (TRS).

O cenário ideal para iniciar os métodos dialíticos é dentro de um planejamento, com seguimento prévio em consultas com equipe multidisciplinar, incluindo o nefrologista, envolvendo orientações sobre a diálise, mudanças de estilo de vida e otimização do tratamento farmacológico e não farmacológico. Além disso, no momento do início da TRS, na hemodiálise (HD), o acesso dialítico por fístula arteriovenosa deve estar confeccionado e funcionante. Na diálise peritoneal (DP), o cateter de Tenckhoff precisa estar cicatrizado e funcionando apropriadamente, o domicílio deve estar adequado para a terapia e os cuidadores ou o paciente treinados para sua realização^2^.

Apesar de ser desejável, essa não é a realidade em boa parte do mundo. No Brasil, aproximadamente 60% dos pacientes não iniciam a diálise de maneira planejada^5,6^. As causas são várias, incluindo diagnóstico tardio, obstáculos na referência ao especialista ou dificuldade na obtenção precoce de acessos definitivos^7,8,9,10,11^.

A diálise não planejada, também chamada de início urgente, por definição, é o início da terapia sem seguimento prévio com nefrologista ou, se realizado, inferior a 90 dias, podendo ser DP ou HD, com as seguintes particularidades: na HD, a TRS inicia-se sem fístula arteriovenosa funcionante e utilizável, portanto através de cateter venoso central (CVC)^12^. Na DP, caracterizar-se-ia pelo início da terapia dentro de 3 dias após o implante do cateter, sem treinamento familiar prévio e sem preparo domiciliar^13^.

No cenário planejado, os resultados clínicos entre DP e HD são bastante semelhantes, principalmente nos dois primeiros anos de terapia^14,15^. Crescentes evidências no cenário não planejado concluem que a DP também é opção segura e eficiente. Uma revisão sistemática de Zang et al.^16^ publicada em 2017 mostrou que pacientes tratados por DP de início urgente apresentaram complicações infecciosas e mortalidade semelhantes aos pacientes tratados por HD de início urgente. Mais recentemente, Ding et al.^17^, em revisão sistemática publicada em 2022, mostraram menor mortalidade e menores taxas de complicações infecciosas nos pacientes tratados por DP de início urgente. Além do que concluem esses estudos, existem vantagens atribuídas à preservação da rede vascular e da função renal residual e menor número de complicações graves, como bacteremia e internações^5,18,19,20,21,22^. Sua utilização é ferramenta importante para aumentar programas de DP^19^, opção para as escassas vagas de HD no país^23,24,25^.

Apesar de desfechos positivos, em terapias de alto custo e impacto orçamentário, resultados clínicos exclusivamente não são suficientes para alteração de políticas de saúde, sendo essencial a realização de análises econômicas. No cenário de TRS planejada, os estudos até então publicados indicam a DP como terapia mais custo-efetiva que a HD^26,27,28^. Entre esses estudos, cita-se o realizado por Chang et al.^27^, o qual avaliou a sobrevida em 14 anos de seguimento e os custos com as terapias HD *vs* DP planejada. A expectativa de vida foi semelhante entre os tratamentos (19,2 *vs* 19,08 anos), assim como a qualidade de vida. Os resultados mostram que a DP foi dominante, com economia de 5 mil dólares por ano de vida ajustados pela qualidade (AVAQ). No entanto, a exclusão de pacientes incidentes^27^ deixa de fora uma importante população, visto que a morbidade e mortalidade nos primeiros meses de terapia é elevada^4^.

No cenário de TRS não planejada, apenas um estudo americano, publicado em 2014^29^, analisou os custos associados aos métodos dialíticos ao longo dos primeiros 90 dias de tratamento, concluindo-se que a DP de início urgente pode ter menores custos (U$ 16.398 comparado a U$ 19.352 para HD de início urgente).

A escassez de vagas de HD no Brasil, frequentemente noticiada pela mídia^23,24,25^, e o custo elevado para sua expansão, tornam a DP uma alternativa interessante de ampliação, e não substituição, da TRS, por não necessitar de grandes estruturas^30^ e maquinários. O estudo é justificado pela evidente necessidade de maiores análises econômicas na TRS não planejada, tendo como objetivo avaliar clínica e economicamente, na perspectiva do SUS, a estratégia de iniciar métodos dialíticos de forma não planejada, com horizonte temporal de doze meses a partir do início da terapia ambulatorial.

## Métodos

Estudo quase experimental com análise econômica através da avaliação de custo-efetividade a partir de dados primários de pacientes incidentes em DP e HD no HC-FMB. Foram coletados dados prospectivamente de todos os pacientes com DRC estágio 5, maiores de 18 anos, cujo início do tratamento dialítico ocorreu necessariamente de forma não planejada no período de 1 de janeiro de 2016 a 1 de janeiro de 2018. O HC-FMB é o único serviço terciário do SUS em sua região, sendo os atendimentos de alta complexidade realizados exclusivamente na instituição. Dessa forma, os dados de atendimento registrados em prontuário são completos e fidedignos.

As indicações imediatas de diálise foram: uremia e casos refratários de hipervolemia, hipercalemia e acidose metabólica. As contraindicações absolutas à DP são: presença de cirurgia abdominal recente (inferior a 30 dias); múltiplas cirurgias abdominais prévias (superior a duas); presença de fibrose ou aderências peritoneais; peritonites fúngicas; insuficiência respiratória severa (FiO^2^ > 70%); infecções de parede abdominal; hipercalemia severa com alterações eletrocardiográficas características e edema agudo de pulmão^31^.

### Alocação nos Grupos e Procedimentos Dialíticos

A aleatorização do início de TRS é tarefa até então sem sucesso na literatura^14^, visto que existem contraindicações absolutas à realização da DP e sempre se deve levar em consideração a escolha do paciente.

Na ausência das contraindicações já citadas, com o consentimento do paciente e dos familiares, o paciente foi alocado no grupo de início por DP e submetido ao implante do cateter de Tenckhoff. O início de diálise ambulatorial ocorre no centro dialisador através de DP intermitente (DPI), realizada através de, ao menos, três sessões semanais diurnas de diálise peritoneal automatizada, com ajustes conforme avaliação clínica e laboratorial da equipe médica, até terem condições de manutenção de terapia dialítica domiciliar ou outro desfecho. Em casos selecionados, antes do início da terapia ambulatorial, em ambiente hospitalar, a DP de alto volume foi utilizada até obtenção de adequado controle metabólico e volêmico, conforme publicado^31^.

Nos casos de contraindicação para a realização da DP ou negativa dos familiares ou paciente, o método inicial foi a HD, com implante de cateter duplo lúmen específico e encaminhamento para início do método ambulatorial assim que possível, através de sessões de quatro horas, três vezes por semana, com ajustes conforme avaliação clínica e laboratorial da equipe médica.

### Seguimento dos Pacientes

Os dados foram coletados diretamente dos prontuários médicos. O primeiro ano de diálise foi escolhido como horizonte temporal por apresentar maior taxa de morbidade e mortalidade^4^, ser pouco estudado e para detalhar a estratégia de iniciar a TRS por diferentes métodos. No período de doze meses, os pacientes podem ter permanecido no método de TRS que iniciaram, podem ter mudado de método de diálise, recuperado função renal, recebido um transplante renal ou até evoluído para óbito. Os dados exatos sobre o tempo em cada estado estavam disponíveis, inclusive se houve mais de uma mudança (exemplo: HD para DP e, depois, transplante). As informações referentes a procedimentos em acessos para diálise, exames de rotina, manutenção da terapia dialítica, internações e fornecimento de medicamentos relacionados à DRC em estágio final (CID N18.0) do Programa de Medicamentos de Dispensação em Caráter Excepcional (medicações de alto custo) foram coletadas exclusivamente dos períodos que os pacientes estiveram em métodos dialíticos, refletindo os custos relacionados a estes.

### Cálculo de ano de Vida Ajustado Pela Qualidade (AVAQ) no Primeiro ano de Terapia

O AVAQ é um desfecho econômico padronizado que combina sobrevida e qualidade. São calculados pela multiplicação da duração de tempo em um determinado estado de saúde pelo peso obtido no escore de utilidade (peso de utilidade) associado àquele estado de saúde^32^. As estimativas de utilidade variam de 0 a 1, sendo 0 estar morto e 1 ter saúde plena. Um ano vivendo, do início ao fim, com um escore de utilidade de 1 (ou seja, em saúde plena), corresponde a um AVAQ.

O atual grupo de trabalho não aplicou escores de utilidade. Dessa forma, a imputação de dados foi baseada na metanálise publicada por Wyld et al.^33^ Foi suposto que todo paciente que apresenta recuperação da função renal permanece em estado semelhante ao portador de DRC em estágio pré-dialítico e que o escore de utilidade do transplante permanece inalterado independentemente do tempo em relação à cirurgia.

Para o cálculo de AVAQs, foi utilizado o método de área sob a curva^34^. Os valores de utilidade foram multiplicados pelo tempo em cada estado (diálise, recuperação de função renal, transplante, óbito) durante o primeiro ano após início de terapia. Assumiu-se que o paciente permaneceu com a mesma utilidade durante todo o tempo que esteve em cada um dos estados.

### Obtenção de Custos

Por metodologia de *bottom-up* ou microcusteio, a coleta de dados ocorreu durante o acompanhamento dos pacientes, diretamente de seus prontuários médicos, obtendo-se dados reais sobre tempo em terapia dialítica, número de sessões de diálise realizadas, medicações administradas, procedimentos em acessos dialíticos realizados e eventos ocorridos, possibilitando cálculo de custos para o financiador através dos valores repassados pelo Sistema Único de Saúde durante os períodos que os pacientes estiveram em diálise pelo tempo de seguimento.

A coorte foi analisada por categorias de custos, sendo elas os custos diretos médicos relacionados às políticas de média e alta complexidade do Sistema Único de Saúde e relacionados ao tratamento dialítico (exames de rotina, terapia dialítica de manutenção, acesso dialítico e medicações do Programa de Medicamentos de Dispensação em Caráter Excepcional em uso relacionados ao CID N18.0) e hospitalizações. Não foram computados os custos diretos médicos não relacionados às políticas de média e alta complexidade do SUS (por exemplo, fornecimento de medicações para controle de pressão arterial e diabetes *mellitus*), medicações do Programa de Medicamentos de Dispensação em Caráter Excepcional em uso não relacionados ao CID N18.0 (por exemplo, medicações para hipercolesterolemia refratária, pós-síndrome coronariana aguda, imunossupressores) e durante períodos em que o paciente não esteve em diálise. Também não foram utilizados para análise os custos diretos não médicos (por exemplo, fornecimento de suplementos e transporte) e os custos indiretos, como benefícios e perda de dias de trabalho (financiamento pela Seguridade Social). Os custos com tratamento ambulatorial de infecções relacionadas ao acesso dialítico não foram incorporados, já que o SUS não oferece repasse específico para essas situações.

Os custos para o SUS com terapias dialíticas, exames de rotina e acessos para diálise foram obtidos pela multiplicação do número de procedimentos de cada paciente pelo valor de repasse dos respectivos procedimentos registrados no Sistema de Gerenciamento da Tabela de Procedimentos, Medicamentos e Órteses, Prótese e Materiais Especiais (OPME) do SUS (SIGTAP). Foram utilizados os valores disponíveis em dezembro de 2019.

Em relação às internações, o custo para o SUS foi obtido através da multiplicação do número de dias que esses pacientes permaneceram internados durante períodos em terapias dialíticas pelo valor do código 03.05.01.017-4 – Tratamento de intercorrência em paciente renal crônico sob tratamento dialítico (por dia) do SIGTAP disponível em dezembro de 2019.

Para cálculo de valor gasto pelo SUS no fornecimento de medicamentos relacionados à DRC em estágio final (CID N18.0) do Programa de Medicamentos de Dispensação em Caráter Excepcional (sevelâmer, alfaepoetina, calcitriol, hidróxido de ferro e cloridrato de cinacalcete), utilizou-se valores de licitações fechadas disponíveis na Bolsa Eletrônica de Compras do Estado de São Paulo (BEC/SP)^35^ disponíveis até 31 de dezembro de 2019. No caso de haver mais de um valor disponível, foi calculada a média dos valores para cada medicação. Os valores foram: sevelâmer: R$ 3,05 por comprimido; alfaepoetina 4.000 U: R$ 18,30/ampola; alfaepoetina 10.000 U: R$ 48,65/ampola; calcitriol 0,25 mcg: R$ 1,05/comprimido; calcitriol 1 mcg: R$ 4.78/ampola; hidróxido de ferro 100 mg: R$ 4.78/ampola; cloridrato de cinacalcete 30 mg: R$ 3,72/comprimido.

### Análise Estatística

A análise do estudo foi por intenção de tratamento, a fim de avaliar os efeitos e custos, no fim de um ano, de iniciar a terapia dialítica não planejada por HD ou DP. A partir do protocolo de estudo, os dados foram inseridos em uma planilha da Microsoft Excel 2013. Foram então verificados erros tipográficos e feita a análise pelo programa estatístico SAS para Windows (versão 9.2: SAS Institute, Cary, NC, EUA, 2012).

Considerando-se erro alfa de 5%, erro beta de 20%, poder estatístico do teste de 80% e detecção de diferença de custo entre os grupos de 15%, o tamanho da amostra calculado para cada grupo foi de 94 pacientes.

A análise descritiva foi realizada para todos os pacientes inseridos no período, calculadas medidas de tendência central e dispersão para variáveis contínuas e frequências para variáveis categóricas. Na comparação entre os grupos, a estatística foi realizada através do teste qui-quadrado para as variáveis categóricas e do teste T ou Mann-Whitney para as variáveis contínuas. Foram criadas as curvas de sobrevida dos pacientes que iniciaram TRS pelos dois métodos durante o período de monitorização, utilizando Kaplan Meyer e *log rank*. A diferença estatística foi considerada significativa quando p < 0,05.

### Análise Econômica

O estudo foi uma análise econômica baseada neste único estudo (*single study-based economic evaluation*), na perspectiva da fonte pagadora, o Sistema Único de Saúde, com horizonte temporal de doze meses a partir do início da TRS ambulatorial de cada paciente. As medições de efetividade e utilidade selecionadas foram, respectivamente, os meses de vida no primeiro ano e os AVAQs.

Havendo diferença dos dados de custos e efetividades, foi proposto o cálculo da razão de custo-efetividade incremental (RCEI) e a análise dos dados por *bootstrapping,* através do processamento dos dados pelo Stata Statistical Software (Versão 14, StataCorp, 2015), obtendo-se 2.000 reamostragens para cada variável. Esse método permite inferências sobre as médias aritméticas em amostras assimétricas ao assumir que a distribuição empírica obtida é uma representação adequada da distribuição dos dados na população real^34^. Com isso, torna-se possível cálculos de intervalos de RCEIs, desenvolvimento de curvas de aceitabilidade, planos de custo-efetividade incremental e interpretação dos resultados baseados em limiares de disposição a pagar (*willingness to pay*). Permite também a realização de análises de sensibilidade determinísticas univariadas para avaliação das incertezas de parâmetros (custos das internações, de medicamentos, exames de rotina e AVAQs) e de cenários hipotéticos de aumento de repasse das terapias dialíticas e acessos para HD e DP, conforme as *Diretrizes metodológicas: Diretriz de Avaliação Econômica* do Ministério da Saúde^36^, com construção de diagrama de tornado pelo Microsoft Excel 2013. A metodologia escolhida substitui de forma mais adequada a construção de modelos econômicos por ser específica para estudos de dados primários, conforme diretrizes sobre o tema^34,37^.

Pelo período de seguimento de cada paciente ser inferior a um ano, a taxa de desconto não é aplicável.

### Aspectos Éticos

A pesquisa foi aprovada pelo Comitê de Ética em pesquisa da instituição do estudo (Parecer 1.951.845/2017) e demais instâncias necessárias com base na Resolução 466/12 do Conselho Nacional de Saúde. Também foi registrado no *ClinicalTrial.gov* (NCT02646436).

## Resultados

Duzentos e oito pacientes foram incluídos no estudo, sendo nove excluídos por dados incompletos e/ou inconsistências e um por ter sido transferido de centro de diálise (perda de dados).

Quanto à composição dos grupos, não houve diferença entre o grupo que iniciou por HD e DP quanto à idade (58,65±15,57 *vs* 59,65±16,17 anos, p = 0,658), sexo masculino (44,4 *vs* 55,6%, p = 0,886) e prevalência de múltiplas comorbidades (81 *vs* 78%, p = 0,592). Os grupos também não divergiram quanto à causa da DRC (Tabela 1).

**Tabela 1:** Características clínicas dos pacientes tratados com diálise não planejada

Características clínicas	Início por HD	Início por DP	Valor de p
	(n = 99)	(n = 99)	
Idade (anos)	58.65 ± 15.57	59,65 ± 16,17	0,658
Sexo (masculino, n, %)	44 (44,4%)	55 (55,6%)	0,886
Duas ou mais comorbidades (n, %)	81 (81,8%)	78 (78,8%)	0,592
Etiologia da DRC (n, %)			0,674
Diabetes *mellitus*	30 (30,3%)	38 (38,4%)	0,295
Hipertensão arterial	19 (19,2%)	16 (16,2%)	0,71
Glomerulopatia	11 (11,1%)	11 (11,1%)	1
Obstrutiva	5 (5,1%)	7 (7,1%)	0,767
Outras	34 (34,3%)	27 (27,3%)	0,356

Nota: Valores expressos em média ± DP ou número (porcentagem).

Em relação ao grupo que iniciou por HD, o grupo que inicia por DP apresentou maior frequência de transição de método dialítico (1% *vs* 24,2%, p < 0,001), refletindo em uma menor porcentagem de pacientes que completaram 12 meses na terapia dialítica que iniciaram (67,7% *vs* 52,5%, p = 0,029). Não houve diferença em relação ao número de transplantes renais (4 *vs* 4%, p = 1,0) e de pacientes que apresentaram recuperação de função renal (2 *vs* 8, p = 0,052). Também, os grupos equipararam-se no número de dias internados [0 (0–9) *vs* 1 (0–8), p = 0,55] e infecções [1 (0–1) *vs* 1 (0–2), p = 0,54], porém o grupo que iniciou por HD necessitou de mais procedimentos relacionados a acessos dialíticos [3 (2–4) *vs* 1 (1–3), p < 0,001] durante o período. A mortalidade (26% *vs* 15%, p = 0,054) e o tempo de sobrevida nos 12 meses [12 (11,51–12) *vs* 12 (12–12), p = 0,063] foram semelhantes entre os dois grupos, mas, quando foi calculada a quantidade de AVAQs obtidos no primeiro ano de terapia utilizando os dados da metanálise de Wyld et al.^33^, foi observado que o grupo que iniciou por DP apresentou vantagem em relação ao grupo que iniciou por HD (0,59 ± 0,21 *vs* 0,65 ± 0,19; p = 0,035) (Tabela 2). A curva de Kaplan-Meier para mortalidade pode ser visualizada na Figura 1.

**Tabela 2:** Desfechos clínicos

Desfechos	Início por HD	Início por DP	Valor de p
	(n = 99)	(n = 99)	
Tempo de sobrevida durante acompanhamento (meses)			
Mediana	12 (11,51–12)	12 (12–12)	0,063
Média*	10,2 ± 3,65	10,84 ± 3,10	0,186
Tempo total em terapias dialíticas durante acompanhamento (meses)	12 (11,9–12)	12 (8,32–12)	0,265
Manutenção do método de diálise em que iniciou	67 (67,7%)	52 (52,5%)	0,029
Desfechos intermediários			
Mudança de método durante seguimento	1 (1%)	24 (24,2%)	< 0,001
Recuperação de função renal durante seguimento	2 (2%)	8 (8,1%)	0,052
Transplante renal durante seguimento	4 (4%)	4 (4%)	1
Óbito no fim de 12 meses	26 (26,3%)	15 (15,2%)	0,054
Procedimentos em acessos	3 (2–4)	1 (1–3)	< 0,001
Dias internado	0 (0–9)	1 (0–8)	0,55
Episódios de infecções relacionadas à diálise	1 (0–1)	1 (0–2)	0,54
AVAQs obtidos no primeiro ano*	0,59 ± 0,21	0,65 ± 0,19	0,035

Nota: Valores expressos em média ± DP, mediana (intervalo interquartil) ou número (porcentagem).

* Dados não paramétricos analisados por métodos paramétricos devido à análise econômica.

**Figura 1:** Curva de Kaplan-Meier para sobrevida em 12 meses a partir do início da diálise ambulatorial.

Em relação aos custos, o gasto médio do SUS com o paciente em diálise durante o primeiro ano de terapia foi semelhante nos dois grupos (R$ 33.081,11 ± 12.760,52 *vs* R$ 35.478,96 ± 12.470,31, p = 0,607). O financiamento de procedimentos dialíticos corresponde à maior fatia do custo e é maior no grupo que inicia por DP (R$ 25.039,5 *vs* R$ 29.380,24, p = 0,002). As medicações do Programa de Medicamentos de Dispensação em Caráter Excepcional são maiores no grupo que inicia por HD (R$ 5.187,78 *vs* R$ 4.103,37, p = 0,018), assim como os gastos com acessos dialíticos (R$ 1.713,04 *vs* R$ 976,57, p < 0,001) e exames de rotina (R$ 616,99 *vs* R$ 535,78, p = 0,01). Quanto aos valores relacionados às internações (R$ 523,78 *vs* R$ 482,99, p = 0,752), não foram observadas diferenças entre os grupos analisados (Tabela 3).

**Tabela 3:** Análise de custos

Custos	Início por HD	Início por DP	Valor de p
	(n = 99)	(n = 99)	
Gastos com paciente durante terapias dialíticas no seguimento	R$ 33.081,11 ± 12.760,52	R$ 35.478,96 ± 12.470,31	0,607
Gastos por categoria			
Terapia dialítica*	R$ 25.039,5 (76%)	R$ 29.380,24 (83%)	0,002
Medicações	R$ 5.187,78 (16%)	R$ 4.103,37 (12%)	0,018
Internações*	R$ 523,78 (2%)	R$ 482,99 (1%)	0,752
Acessos dialíticos*	R$ 1.713,04 (5%)	R$ 976,57 (3%)	< 0,001
Exames de rotina*	R$ 616,99 (2%)	R$ 535,78 (2%)	0,01
Custo por mês de vida no primeiro ano*	R$ 3.462,89 ± 1.145,54	R$ 3/350,81 ± 759,03	0,418
Custo por AVAQ*	R$ 60.071,62 ± 19.700,96	R$ 56.090,17 ± 13.022,79	0,095

Nota: Valores expressos em média ± DP ou valor (porcentagem do total de gastos)

* Dados não paramétricos analisados por métodos paramétricos devido análise econômica.

Dessa forma, iniciar por DP ou HD não difere em relação a custos e efetividade (meses de vida no primeiro ano), podendo haver utilidade melhor no grupo que inicia por DP. Ao se analisar o gasto relacionado ao paciente em diálise por mês de vida no primeiro ano (R$ 3.462,89 ± 1.145,54 *vs* R$ 3.350,81 ± 759,03, p = 0,418) e o gasto relacionado ao paciente em diálise por AVAQ obtido no primeiro ano (R$ 60.071,62 ± ١٩.٧٠٠,٩٦ *vs* R$ 56.090,17 ± 13.022,79, p = 0,095) não se notam diferenças. Devido à ausência de diferenças em custos e efetividade, as terapias são tidas como equivalentes e os cálculos da RCEI e análises pormenorizadas não têm utilidade (Tabela 3). O grupo optou por interpretar com cautela os resultados de utilidade e não prosseguir em análises mais avançadas devido a dados extrapolados de estudo de outra população, conforme pormenorizado na discussão.

## Discussão

Ao se discutir sobre a realidade da DRC em estágios mais avançados, dois pontos são marcantes em países em desenvolvimento: a falta de acesso às terapias renais substitutivas^38,39^ e o início de terapia dialítica de maneira não planejada^5,6,7,8,9,40,41^. Em cenário de recursos limitados, os custos impactam diretamente a morbidade e mortalidade dos pacientes com necessidade desse tipo de tratamento^4,42^. A DP é opção terapêutica com potencial de alterar essa realidade, porém permanece subutilizada^6,10,11^ por fatores que vão desde desconhecimento técnico a conceitos ultrapassados baseados em estudos antigos que traziam dúvidas quanto aos resultados da DP.

O estudo mostrou que, no cenário da TRS não planejada, iniciar por diálise peritoneal foi equivalente a iniciar por HD, com custos e efetividade semelhantes. O resultado de mais AVAQs no primeiro ano ao iniciar por DP, inferindo melhor utilidade, apesar de positivo e animador, não foi valorizado a ponto de ser incluído em uma análise econômica pormenorizada, por ser a variável de maior viés do trabalho. Os dados de utilidade foram retirados de uma metanálise internacional^33^ e não da população do estado ou da população brasileira.

Vale ressaltar ainda que, além das vantagens clínicas já citadas, a DP apresenta fatores políticos e econômicos relevantes não abordados neste estudo. Ao se optar por uma política de estímulo à DP, há uma notável economia com gastos em infraestrutura. Por ser uma terapia realizada no domicílio do paciente e não dentro do centro dialisador, não há exigência de construção ou adequação de grandes estruturas prediais nem de aquisição de maquinário específico de alto custo. Na questão predial, pela Resolução da Diretoria Colegiada (RDC) 50 de 2002^30^, uma unidade exclusivamente de diálise peritoneal pode ser credenciada e iniciada em unidades de saúde regulares com a RDC, públicas ou particulares, hospitalares ou ambulatoriais, muitas vezes sem necessidade de aquisição de materiais ou até de reformas, caso haja salas vagas compatíveis com as poucas exigências. A título de ilustração, isso poderia ser facilmente adequado em clínicas particulares com consultórios vagos ou, inclusive, em unidades básicas de saúde com salas ociosas. Para a instalação de um centro de hemodiálise, minimamente, considerando uma unidade que não faz reprocessamento de capilares e sem sala para pacientes com hepatite B, além de um grande salão ou múltiplas salas pequenas para as poltronas e/ou os leitos de hemodiálise, seria necessário um recinto para tratamento e reservatório de água tratada para diálise, área de lavagem de fístulas e adequações proporcionais ao número de poltronas e/ou leitos ao menos em relação ao posto de enfermagem e serviços e à sala de recuperação de pacientes. Em relação ao maquinário, o regime frequentemente adotado pelos fornecedores de materiais para DP é o de comodato, com fornecimento da máquina de DPA sem ônus para a unidade executante, incluindo a manutenção^43^. Por mais que as máquinas de hemodiálise também possam ser fornecidas em regime de comodato ou aluguel, diluindo os gastos iniciais, é necessária a aquisição de cadeiras e/ou leitos e de um sistema central de tratamento e distribuição de água, cujo valor era superior a 260 mil reais em 2022^44,45^.

A necessidade de recursos humanos, energéticos e hídricos também é inferior, assim como de regulamentações, licenças e controles de qualidade^46,47^. Também, na questão logística, não há necessidade do transporte de pacientes habitualmente três vezes por semana para realizar as sessões. Essas características são importantes, principalmente em países em desenvolvimento, ao se avaliar a expansão de vagas de diálise. No Brasil, esse problema crônico é esporadicamente noticiado pela mídia, quando, no caso da indisponibilidade de vagas ambulatoriais de HD, os pacientes que necessitam iniciar TRS por HD o fazem durante internação e permanecem em leitos hospitalares até a disponibilização, o que pode demorar semanas ou até meses^23,24,25^.

O estudo tem pontos fortes, a se destacar a utilização de dados primários, reduzindo incertezas, inferências e, consequentemente, a necessidade de correções. As informações extraídas diretamente dos prontuários eletrônicos dos pacientes trazem solidez e confiabilidade aos dados em todos os períodos, inclusive durante mudanças de método. A característica pragmática do estudo, com dados de mundo real, permite uma validação externa mais fácil, apesar da análise primária e de ser um estudo unicêntrico. A opção de estudo por intenção de tratamento torna a avaliação clínica e econômica mais fluida e conclusiva, pois não se restringe à comparação de dois métodos dialíticos, mas sim da estratégia de se iniciar por um ou por outro; avalia-se uma política de saúde pública.

Limitações, no entanto, estão presentes. Inicialmente, é um estudo unicêntrico. Como exemplo, vale ressaltar que a população estudada tem prevalência de uso de cateter duplo lúmen como acesso dialítico para hemodiálise maior que 60%^19,48^, dados bem superiores à relatada pelo Censo Brasileiro de Diálise de 2022, que mostra prevalência de 20,9% de acessos de longa permanência e 8,3% de acessos de curta permanência entre pacientes em HD^10^. Essa característica pode justificar o maior número de intervenções em acesso dialítico e pode ter interferido na mortalidade do grupo HD, a qual atingiu níveis próximos aos de significância estatística. Outro fator seria o horizonte temporal limitado. A opção do grupo em não construir modelos econômicos a fim de estendê-lo justifica-se para detalhar os eventos e custos do ano de acesso à TRS não planejada, o qual tem maior morbidade e mortalidade, permitindo discutir políticas de saúde pública. Não foi realizada aleatorização devido às dificuldades já citadas, o que pode resultar em vieses, especialmente de seleção, com pacientes em estado de saúde melhor sendo encaminhados para o grupo de diálise peritoneal, apesar da ausência de diferenças entre os grupos estudados. Também, as análises e contabilizações de eventos e custos foram feitas apenas durante os períodos em que o paciente esteve em diálise, excluindo-se dados de outros períodos, com exceção do tempo. A perspectiva do estudo foi do SUS em sua esfera federal através dos repasses no momento do estudo, o que não reflete os reais custos das unidades prestadoras e não necessariamente são proporcionais aos repasses atuais. Há também as já citadas e comentadas críticas quanto aos escores de utilidade utilizados.

O tempo de seguimento e a opção pela análise por intenção de tratamento dificultam a comparação com outras análises econômicas de diálise não planejada, como a de Liu et al.^29^, a qual mostrou custos inferiores, talvez por não levar em consideração a alta porcentagem de transição de método para hemodiálise com o passar dos meses, valores que foram incluídos neste estudo.

Propostas como programas de DP de início não planejada são comprovadamente efetivas^5,19,49,50,51^, porém o aumento da utilização do método envolve questões econômicas, políticas e culturais. No caso da experiência da Unidade de Diálise do HC-FMB, o crescimento do programa de DP não planejada permitiu que um grande contingente de pacientes obtivesse acesso ao tratamento ambulatorial, evitando internações para aguardar vagas ambulatoriais de diálise e, possivelmente, óbitos antes de chegar ao serviço de referência.

## Conclusão

Os resultados mostram que iniciar TRS por DP é opção semelhante a iniciar por HD em pacientes com necessidade de métodos não planejados, com desfechos e custos de manutenção de paciente em TRS semelhantes. No entanto, o baixo investimento inicial em infraestrutura e o maquinário necessário para criação de vagas de DP são fatores que a fortalece como política de saúde pública para expansão da TRS em países em desenvolvimento. São necessários estudos com dados primários sobre qualidade de vida para avaliar mais adequadamente a superioridade em AVAQs a iniciar por DP em relação a iniciar por HD e, consequentemente, a custo-utilidade.

## Material Suplementar

O seguinte material on-line está disponível para o presente artigo:

Tabela s1 - Repasse do SUS para a manutenção de terapias renais substitutivas dialíticas

Tabela s2 - Protocolo de exames segundo o Ministério da Saúde

Tabela s3 - Repasse do SUS para exames protocolares

Tabela s4 - Repasse do SUS para criação e intervenção em acessos dialíticos

Tabela s5 - Valores das medicações segundo a Bolsa de Compras do Estado de São Paulo
